# Modification of the 1-Phosphate Group during Biosynthesis of Capnocytophaga canimorsus Lipid A

**DOI:** 10.1128/IAI.01006-15

**Published:** 2016-01-25

**Authors:** Francesco Renzi, Ulrich Zähringer, Courtney E. Chandler, Robert K. Ernst, Guy R. Cornelis, Simon J. Ittig

**Affiliations:** a Université de Namur, Namur, Belgium; b Division of Immunochemistry, Research Center Borstel, Leibniz Center for Medicine and Biosciences, Borstel, Germany; c Department of Microbial Pathogenesis, University of Maryland, Baltimore, Maryland, USA; d Biozentrum der Universität Basel, Basel, Switzerland

## Abstract

Capnocytophaga canimorsus, a commensal bacterium of dog's mouth flora causing severe infections in humans after dog bites or scratches, has a lipopolysaccharide (LPS) (endotoxin) with low-inflammatory lipid A. In particular, it contains a phosphoethanolamine (*P*-Etn) instead of a free phosphate group at the C-1 position of the lipid A backbone, usually present in highly toxic enterobacterial Gram-negative lipid A. Here we show that the C. canimorsus genome comprises a single operon encoding a lipid A 1-phosphatase (LpxE) and a lipid A 1 *P*-Etn transferase (EptA). This suggests that lipid A is modified during biosynthesis after completing acylation of the backbone by removal of the 1-phosphate and subsequent addition of an *P*-Etn group. As endotoxicity of lipid A is known to depend largely on the degree of unsubstituted or unmodified phosphate residues, deletion of *lpxE* or *eptA* led to mutants lacking the *P*-Etn group, with consequently increased endotoxicity and decreased resistance to cationic antimicrobial peptides (CAMP). Consistent with the proposed sequential biosynthetic mechanism, the endotoxicity and CAMP resistance of a double deletion mutant of *lpxE-eptA* was similar to that of a single *lpxE* mutant. Finally, the proposed enzymatic activities of LpxE and EptA based on sequence similarity could be successfully validated by mass spectrometry (MS)-based analysis of lipid A isolated from the corresponding deletion mutant strains.

## INTRODUCTION

Some Gram-negative bacteria have evolved different modifications of their lipid A structure, leading to a reduced recognition by the host and sensitivity to cationic antimicrobial peptides (CAMP) (1–7). One of these modifications occurs on the 1- or 4′-phosphate of lipid A (1, 4, 7–10). 4′-Phosphatases (LpxF) have been reported in Rhizobium leguminosarum, Rhizobium etli, Porphyromonas gingivalis, Francisella species, and Helicobacter pylori (1, 10–12). Deletion of *lpxF* and the resulting presence of the 4′-phosphate on lipid A leads to increased endotoxicity (1, 12) and decreased resistance to CAMP (10, 12). In the case of Francisella and H. pylori, virulence is reduced (11, 12, 13). 1-Phosphatases (LpxE) have been identified in H. pylori, P. gingivalis, *R. etli*, and others (1, 6, 10, 12, 14–16). Deletion of *lpxE* and the resulting presence of the 1-phosphate on lipid A leads to a slightly increased endotoxicity (1) and CAMP sensitivity (10). In H. pylori, position 1 is further modified by the addition of a phosphoethanolamine (*P*-Etn) (15, 17, 18), a modification known from other bacteria (15, 17, 18). This happens via a two-step mechanism, which first involves dephosphorylation of one phosphate residue located at position C-1 of the lipid A backbone by LpxE and subsequent *P*-Etn transfer by a phosphoethanolamine transferase (EptA or PmrC) (15, 16). In H. pylori, *lpxE* and *eptA* are contained in one operon (Hp0021-Hp0022) (16).

**FIG 1:**
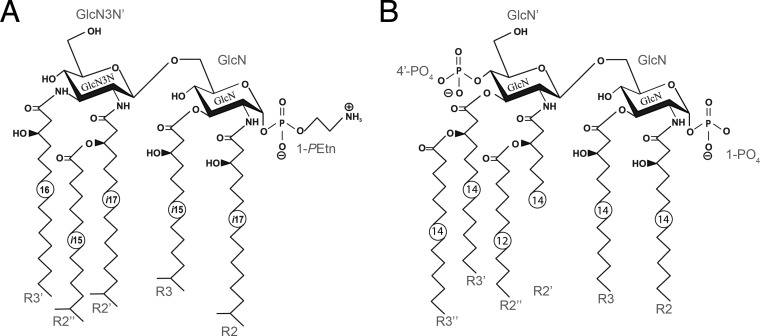
Structures of C. canimorsus strain 5 and E. coli lipid A. (A) C. canimorsus strain 5 lipid A consists of a β-(1′→6)-linked GlcN3N′-GlcN disaccharide, to which 3-hydroxy-15-methylhexadecanoic acid, 3-hydroxy-13-methyltetradecanoic acid, 3-*O*-(13-methyltetradecanoyl)-15-methylhexadecanoic acid, and 3-hydroxyhexadecanoic acid are attached at positions 2, 3, 2′, and 3′, respectively. The disaccharide carries a positively charged ethanolamine at the 1-phosphate and lacks a 4′-phosphate (19). (B) E. coli hexa-acylated lipid A consisting of a β-(1′→6)-linked GlcN disaccharide that is phosphorylated at positions 1 and 4′ and carries four (*R*)-3-hydroxymyristate chains (at positions 2′, 3′, 2, and 3). The 2′ and 3′ 3-hydroxylated acyl groups in GlcN′ are further esterified with laurate and myristate, respectively (29).

We have previously characterized the lipid A structure of Capnocytophaga canimorsus (19), a bacterial species that can cause rare but severe sepsis or meningitis in humans after dog bites or scratches (20–24). C. canimorsus belongs to the family Flavobacteriaceae in the phylum Bacteroidetes and is a usual member of dog's mouth flora (21, 25–28). C. canimorsus lipid A consists of a 2,3-diamino-2,3-dideoxy-d-glucose (GlcN3N′) β-(1′→6)-linked to 2-amino-2-deoxy-d-glucose (GlcN) [β-d-Glc*p*N3N′-(1→6)-d-Glc*p*N lipid A hybrid backbone] containing an *P*-Etn group attached to the C-1 reducing end and lacking a 4′-phosphate (Fig. 1A). 3-Hydroxy-15-methylhexadecanoic acid [*i*17:0(3-OH)], 3-hydroxy-13-methyltetradecanoic acid [*i*15:0(3-OH)], 3-*O*-(13-methyltetradecanoyl)-15-methylhexadecanoic acid [*i*17:0[3-*O*(*i*15:0)]], and 3-hydroxyhexadecanoic acid [16:0(3-OH)] are attached to the backbone at positions 2, 3, 2′, and 3′, respectively (19). This structure differs from that of a potent Toll-like receptor 4 (TLR4) agonist like the Escherichia coli lipid A (Fig. 1B), consisting of a β-(1′→6)-linked GlcN disaccharide that is phosphorylated at positions 1 and 4′ and carries four (*R*)-3-hydroxymyristate chains [14:0(3-OH)] (at positions 2′, 3′, 2, and 3). The 2′ and 3′ 3-hydroxylated acyl groups in GlcN(II) are further esterified with laurate and myristate, respectively (29).

We have identified *lpxE* and *eptA* genes in the genome of C. canimorsus and found the overlapping genes to be organized in one operon. We show that the deletion of *lpxE* or *eptA* leads to increased endotoxicity and decreased resistance to CAMP, where deletion of *lpxE* has a more severe effect. Interestingly, the endotoxicity and CAMP resistance of a double deletion mutant of *lpxE* and *eptA* were the same as those of a single *lpxE* mutant. This suggests that the *P*-Etn-containing lipid A is synthesized by a similar two-step enzymatic process as in H. pylori, where dephosphorylation is necessary for substitution of 1-phosphate with *P*-Etn. Finally, we could successfully validate the proposed lipid A structures of the respective deletion mutants by mass spectrometry (MS) analysis, thus also further confirming, on a structural basis, the proposed enzymatic activities of LpxE and EptA as well as the two-step enzymatic mechanism in the lipid A biosynthesis.

## MATERIALS AND METHODS

### Bacterial strains and growth conditions.

The bacterial strains used in this study are listed in Table 1. Escherichia coli strains were grown in LB broth at 37°C. Capnocytophaga canimorsus strain 5 (*Cc*5) (30) was routinely grown on heart infusion agar (HIA; Difco) supplemented with 5% sheep blood (Oxoid) for 2 days at 37°C in the presence of 5% CO_2_. Bacteria were harvested by scraping colonies off the agar surface, washed, and resuspended in phosphate-buffered saline (PBS). The following selective agents were added at the concentrations indicated: erythromycin, 10 μg/ml; cefoxitin, 10 μg/ml; gentamicin, 20 μg/ml; ampicillin, 100 μg/ml; tetracycline, 10 μg/ml.

**TABLE 1:** Bacterial strains and plasmids used in this study

Strain and genotype or plasmid	Origin, construction, description, or relevant genotype and/or phenotype	Reference
Strains		
*Cc*5	Isolated from a case of human fatal septicemia after a dog bite in 1995	30
*Cc*5 Δ*lpxE*	Replacement of *Ccan_16960* by *ermF*; Em^r^ (primers 6493 to 6498) (Δ 1833737-1833995)	This study
*Cc*5 Δ*eptA*	Replacement of *Ccan_16950* by *ermF*; Em^r^ (primers 6499 to 6504) (Δ 1831370-1832888)	This study
*Cc*5 Δ*lpxE-eptA*	Replacement of *Ccan_16960-16950* by *ermF*; Em^r^ (primers 6493 to 6495 and 6502 to 6504) (Δ 1831370-1833995)	This study
*Cc*5 Y1C12	Tn*4351* insertion in *Ccan_23370*, “*wbuB*”-like glycosyltransferase	35
*Cc*5 Y1C12 Δ*lpxE*	Replacement of *Ccan_16960* by *tetQ*; Tc^r^ (primers 7539 to 7544) (Δ 1833737-1833995)	This study
*Cc*5 Y1C12 Δ*eptA*	Replacement of *Ccan_16950* by *tetQ*; Tc^r^ (primers 7545 to 7550) (Δ 1831370-1832888)	This study
*Cc*5 Y1C12 Δ*lpxE-eptA*	Replacement of *Ccan_16960-16950* by *tetQ*; Tc^r^ (primers 7539, 7540, 7543, 7547, 7548, and 7550) (Δ 1831370-1833995)	This study
Plasmids		
p-*lpxE*	pMM47.A*lpxE* (expression plasmid carrying the complete *lpxE* gene from *Cc*5)	This study
p-*lpxE-eptA*	pMM47.A*lpxE-eptA* (expression plasmid carrying the complete *lpxE-eptA* genes from *Cc*5)	This study
p-*eptA*	pMM47.A*eptA* (expression plasmid carrying the complete *eptA* gene from *Cc*5)	This study
pMM13	ColE1 *ori*; Apr (Em^r^); *ermF* from pEP4351	31
pMM25	ColE1 *ori*; Km^r^ (Cf^r^); suicide vector for C. canimorsus	31
pMM47.A	ColE1 *ori* (pCC7 *ori*); Ap^r^ (Cf^r^); E. coli-C. canimorsus expression shuttle plasmid. C. canimorsus expression is driven by an *ermF* promoter.	31
pMM104.A	ColE1 *ori* (pCC7 *ori*); Ap^r^ (Tc^r^); E. coli-C. canimorsus shuttle plasmid, RP4 oriT. The PstI fragment of pMM47.A containing *repA* was inserted into the PstI site of pLYL001.	31
pSI73	pMM25*lpxE*::*ermF* (suicide vector for deletion of *lpxE* [Δ 1833737-1833995])	This study
pSI74	pMM25*eptA*::*ermF* (suicide vector for deletion of *eptA* [Δ 1831370-1832888])	This study
pSI76	pMM25*lpxE-eptA*::*ermF* (suicide vector for deletion of *lpxE-eptA* [Δ 1831370-1833995])	This study
pFR28	pMM25*lpxE*::*tetQ* (suicide vector for deletion of *lpxE* [Δ 1833737-1833995])	This study
pFR29	pMM25*eptA*::*tetQ* (suicide vector for deletion of *eptA* [Δ 1831370-1832888])	This study
pFR30	pMM25*lpxE-eptA*::*tetQ* (suicide vector for deletion of *lpxE-eptA* [Δ 1831370-1833995])	This study

### Genetic manipulations of C. canimorsus.

Genetic manipulations of wild-type (wt) *Cc*5 have been described (31). Briefly, replacement cassettes with flanking regions spanning approximately 500 bp homologous to regions directly adjacent to the *lpxE* or *eptA* gene (28) were constructed with a three-fragment overlapping PCR strategy. As the ATG codon of the *eptA* gene is located within the coding region of *lpxE*, 106 bp upstream of the *eptA* ATG codon was not deleted in an *lpxE* single knockout (Δ 1833737-1833995). First, two PCRs were performed on 100 ng of *Cc*5 genomic DNA with primers A and B (Table 2) for the upstream flanking regions and with primers E and F for the downstream regions. Primers B and E contained an additional 5′ 20-nucleotide extension homologous to the *ermF* or *tetQ* insertion cassette. The *ermF* and *tetQ* resistance cassettes were amplified from plasmids pMM13 and pMM104.A DNA, respectively, with primers C and D. All three PCR products were cleaned and then mixed in equal amounts for PCR using Phusion polymerase (Finnzymes). The initial denaturation was at 98°C for 2 min, followed by 12 cycles without primers to allow annealing and elongation of the overlapping fragments (1 cycle consists of 98°C for 30 s, 50°C for 40 s, and 72°C for 2 min). After the addition of external primers (primers A and F), the program was continued with 20 cycles (1 cycle consists of 98°C for 30 s, 50°C for 40 s, and 72°C for 2 min 30 s) and finally 10 min at 72°C. Final PCR products containing *lpxE*::*ermF*, *eptA*::*ermF*, *lpxE-eptA*::*ermF*, *lpxE*::*tetQ*, *eptA*::*tetQ*, or *lpxE-eptA*::*tetQ* insertion cassettes were then digested with PstI and SpeI for cloning into the appropriate sites of the C. canimorsus suicide vector pMM25 (31). The resulting plasmids were transferred by RP4-mediated conjugative DNA transfer from E. coli S17-1 to C. canimorsus strain 5 or C. canimorsus strain 5 Y1C12 mutant to allow integration of the insertion cassette. Transconjugants were then selected for the presence of the *ermF* or *tetQ* cassette on erythromycin- or tetracycline-containing plates, respectively, and checked for sensitivity to cefoxitin. Deletion of the appropriate regions was verified by PCR.

**TABLE 2:** Oligonucleotides used in this study

Oligonucleotidereference no.	Oligonucleotide name^*a*^	Sequence (5′-3′)	Restriction site	Gene	PCR primer order
6493	lpxE-A	CCCTGCAGGGCACGTTCGTACCAGTTA	PstI	*lpxE*	A
6494	lpxE-B	GAGTAGATAAAAGCACTGTTATTTGCTTATTTTGAATATTTCGG		*lpxE*	B
6495	lpxE-C	CTTATATTTGCCGCCGAAATATTCAAAATAAGCAAATAACAGTGCTTTTATCTACTCCGATAGCTTC		*ermF*	C
6496	lpxE-D	CTTGCATTATCTTAACACTCATAAAAACAACACTCCCCTACGAAGGATGAAATTTTTCAGGGACAAC		*ermF*	D
6497	lpxE-E	AAAAATTTCATCCTTCGTAGGGGAGTGTTGTTTTTATGAGTGTT		*lpxE*	E
6498	lpxE-F	CAACTAGTAAACCGTTTCAGTTTGGGT	SpeI	*lpxE*	F
6499	eptA-A	CCCTGCAGTGTTCCTCGCCCTGTTAC	PstI	*eptA*	A
6500	eptA-B	GAGTAGATAAAAGCACTGTTTTATTGATTTTTTTTAACATAAAATTTTATC		*eptA*	B
6501	eptA-C	GTTGTACTTAATGATAAAATTTTATGTTAAAAAAAATCAATAAAACAGTGCTTTTATCTACTCCGATAGCTTC		*ermF*	C
6502	eptA-D	ATCTTGTAAATTACGGATTGGTCATTCAATAATTCTACGAAGGATGAAATTTTTCAGGGACAAC		*ermF*	D
6503	eptA-E	AAAAATTTCATCCTTCGTAGAATTATTGAATGACCAATCCG		*eptA*	E
6504	eptA-F	CAACTAGTTCCACCTCATTGAGATTCAC	SpeI	*eptA*	F
6646	p-lpxE-fw	CGTACCATGGTTTTTAAAGAATCAGCAAATAACC	NcoI	*lpxE*	
6647	p-lpxE-rev	CAGTTCTAGATTATTGATTTTTTTTAACATAAAATTTTATC	XbaI	*lpxE*	
6648	p-eptA-fw	CGTACCATGGGATTAAAAAAAATCAATAAATGGACTAACA	NcoI	*eptA*	
6649	p-eptA_rev	GCTTCTCGAGTTAGTCAAAAATGCTCATTTGC	XhoI	*eptA*	
7539	lpxEtetKO-A	GGCTGCAGTTTCCATTCCTTTGGCACGTTCG	PstI	*lpxE*	A
7540	lpxEtetKO-B	CAAAATCAAATGTTAAAAAAAAATTTGCTTATTTTGAATATTTCGGC		*lpxE*	B
7543	lpxEtetKO-C	GCCGAAATATTCAAAATAAGCAAATTTTTTTTTAACATTTGATTTTG		*tetQ*	C
7544	lpxEtetKO-D	GATTTTTTTTAACATAAAATTTTATCTTATTTTGATGACATTGATTTTTGG		*tetQ*	D
7541	lpxEtetKO-E	CCAAAAATCAATGTCATCAAAATAAGATAAAATTTTATGTTAAAAAAAATC		*lpxE*	E
7542	eptAtetKO-F	GGACTAGTCAAGGTAAAGCCAATGTTAAGC	SpeI	*lpxE*	F
7545	eptAtetKO-A	GGCTGCAGTATGGGGAGGAAAGCGTCAATATTG	PstI	*eptA*	A
7546	eptAtetKO-B	CAAAATCAAATGTTAAAAAAAAGCGGTACATTGTTAGTCCATTTATTG		*eptA*	B
7549	eptAtetKO-C	CAATAAATGGACTAACAATGTACCGCTTTTTTTTAACATTTGATTTTG		*tetQ*	C
7550	eptAtetKO-D	CGGATTGGTCATTCAATAATTTTATTTTGATGACATTGATTTTTGG		*tetQ*	D
7547	lpxEtetKO-E	CCAAAAATCAATGTCATCAAAATAAAATTATTGAATGACCAATCCG		*eptA*	E
7548	eptAtetKO-F	GGACTAGTCATTAAGTGCTACCCCTATCTTATC	SpeI	*eptA*	F

a In the oligonucleotide name (e.g., lpxE-A), the target gene is shown first and the PCR primer order (A, B, C, D, E, or F) is shown second. Forward and reverse orientations are indicated by fw and rev, respectively, at the end of the oligonucleotide name. tetKO refers to a knockout of the corresponding gene by introduction of a tetracycline resistance gene.

### Construction of complementation plasmids.

Plasmid pMM47.A was used for expression of LpxE and EptA (31). Full-length *lpxE*, *eptA*, or *lpxE-eptA* genes were amplified with the specific primers listed in Table 2 and cloned into plasmid pMM47.A using NcoI and XbaI or NcoI and XhoI restriction sites, leading to the insertion of a glycine at position 2. Ligated plasmids were cloned in E. coli TOP10.

### Human TLR4 activation assay.

HEK293 cells stably expressing human Toll-like receptor 4 (hTLR4), myeloid differentiation factor 2 (MD-2), cluster of differentiation antigen 14 (CD14), and a NF-κB-dependent reporter (secreted embryonic alkaline phosphatase) were from InvivoGen (HekBlue human TLR4). Growth conditions and endotoxicity assay were as recommended by the supplier (InvivoGen). Briefly, the desired amounts of heat-killed bacteria were placed in a total volume of 20 μl (diluted in PBS) and distributed in a flat-bottom 96-well plate (BD Falcon). A total of 25,000 HekBlue human TLR4 cells in 180 μl were then added to each well, and the plate was incubated for 20 to 24 h at 37°C and 5% CO_2_. Detection of the secreted phosphatase followed the QUANTI-Blue protocol (InvivoGen). The challenged cells (20 μl) were incubated with 180 μl detection reagent (QUANTI-Blue; InvivoGen). The plates were incubated at 37°C and 5% CO_2_, and absorbance was measured at 655 nm using a spectrophotometer (Bio-Rad).

### Polymyxin B sensitivity assay.

Polymyxin B sulfate was obtained from Sigma-Aldrich. The MIC was determined by the agar dilution method based on the CLSI/NCCLS recommendations (32). Briefly, 10^4^ bacteria contained in 2 μl PBS were spotted on HIA plates containing 5% sheep blood and polymyxin B ranging from 0.5 mg/liter to 1,024 mg/liter (2-fold increase per step). The plates were incubated and examined for growth of visible colonies after 48 h and 72 h.

### Genome annotation.

The *Blast*-p search tool (33) against the C. canimorsus 5 genome (28) was used. Search sequences were obtained from the National Center for Biotechnology Information. All available Bacteroidetes group sequences were used in the search, but standard E. coli sequences have also been included. The highest scoring subjects over all the searches have been annotated as corresponding enzymes. Difficulties in annotation were observed only for *lpxE*. The *lpxE* search was based on *lpxF* and/or *lpxE* protein sequences from Porphyromonas gingivalis (1), Francisella novicida (7), Rhizobium etli (10), Helicobacter pylori (12, 16) and on all available Bacteroidetes group *pgpB* protein sequences.

### Preparation of bacteria for LPS extraction.

Compositional analysis of the lipopolysaccharide (LPS) from the wt C. canimorsus 5 strain previously showed that it was highly contaminated with glucose from amylopectin, flavolipin, and capnin, known to be present in Capnocytophaga spp. and Flavobacteriaceae (34). In contrast, the LPS from the C. canimorsus 5 Y1C12 mutant (35) was devoid of such contaminating material. Since compositional analysis of the lipid A and LPS core obtained from the wt strain LPS and that of the Y1C12 mutant revealed no differences with respect to their sugars and fatty acids (19, 34), the Y1C12 mutant was chosen as the background strain to isolate and analyze the lipid A of Δ*eptA*, Δ*lpxE*, and Δ*lpxE-eptA* deletion mutants in detail by MS analysis. While the Y1C12 mutant was chosen as the background strain for MS analysis, please note that human TLR4 activation assays and polymyxin B sensitivity analysis are based on C. canimorsus 5 Δ*eptA*, Δ*lpxE*, and Δ*lpxE-eptA* deletion mutants and complemented mutants based on these deletion mutants. The C. canimorsus 5-based Y1C12 mutant has a transposon insertion within a predicted glycosyltransferase-encoding gene, probably the *N*-acetyl fucosamine transferase WbuB, necessary for the formation of the O antigen (35). Endotoxicity of resulting C. canimorsus 5 Y1C12 Δ*eptA*, Δ*lpxE*, and Δ*lpxE-eptA* deletion mutants was assessed, and the results confirmed results obtained with C. canimorsus 5 Δ*eptA*, Δ*lpxE*, and Δ*lpxE-eptA* deletion mutants (data not shown). C. canimorsus bacteria were harvested from 25 blood plates in PBS, followed by centrifugation at 18,000 × *g* for 30 min. Bacteria were resuspended in cold acetone, incubated with shaking, resuspended in PBS containing 0.5% phenol for killing, again harvested by centrifugation, washed with PBS, and resuspended in 1 ml water. One tenth of the volume was plated on appropriate growth plates to ensure complete bacterial killing. Bacteria were air dried prior to LPS extraction.

### Purification and isolation of free lipid A suitable for MS analysis.

Lipid A was isolated from lyophilized C. canimorsus cell pellets following the extraction method of El Hamidi et al. (36). Briefly, pellets were dissolved in 70% isobutyric acid and 1 M ammonium hydroxide and incubated at 100°C for 1 h. Four hundred microliters of water was added to each sample, and the samples were snap-frozen on dry ice and lyophilized overnight. The samples were then washed twice with 1 ml methanol and reconstituted in 150 μl chloroform-methanol-water (3:1.5:0.25, vol/vol/vol).

### MS-based structural analysis.

Lipid A structures were assessed by negative- and positive-ion matrix-assisted laser desorption ionization–time of flight mass spectrometry (MALDI-TOF MS). Lyophilized lipid A was extracted in chloroform-methanol, and then 1 μl was mixed with 1 μl of norharmane MALDI matrix. All MALDI-TOF MS experiments were performed using a Bruker Microflex MALDI-TOF mass spectrometer (Bruker Daltonics, Billerica, MA). Each spectrum was an average of 300 shots. Electrospray (ES) tuning mix (Agilent, Palo Alto, CA) was used for calibration. Data were analyzed using Bruker Daltonik flexAnalysis software.

### Immunoblotting of proteinase K-resistant structures.

Bacteria were harvested from blood agar plates, washed once in 1 ml of PBS, and adjusted to an optical density at 600 nm (OD_600_) of 1.5 in PBS. Five hundred microliters of bacterial suspension was pelleted and dissolved in 125 μl loading buffer (1% sodium dodecyl sulfate [SDS], 10% glycerol, 50 mM dithiothreitol, 0.02% bromophenol blue, 45 mM Tris [pH 6.8] in double-distilled water [ddH_2_O]). Samples were boiled at 99°C for 10 min. Proteinase K (final concentration of 50 μg/ml) was added, and samples were incubated at 37°C overnight. After incubation, samples were boiled again for 10 min at 99°C, and a second volume of proteinase K (equal to the first) was added. The samples were incubated at 55°C for 3 h, boiled again for 5 min at 99°C, and loaded on a 15% SDS-polyacrylamide gel. The samples were analyzed by Western blotting using polyclonal, C. canimorsus 5 Y1C12-absorbed serum against C. canimorsus 5. This antibody was generated from rabbits by immunization with heat-killed C. canimorsus 5 (Laboratoire d'Hormonologie, Marloie, Belgium). The C. canimorsus 5 Y1C12-absorbed serum was prepared by incubating twice an excess amount of Y1C12 mutant C. canimorsus 5 bacteria (harvested from blood agar plates and washed in PBS) with anti-C. canimorsus 5 serum at 4°C for 12 h. Bacteria were removed by repeated centrifugation. This results in an antiserum recognizing C. canimorsus 5 LPS (35).

## RESULTS AND DISCUSSION

### Identification of enzymes leading to the presence of 1 *P*-Etn on lipid A.

**FIG 2:**
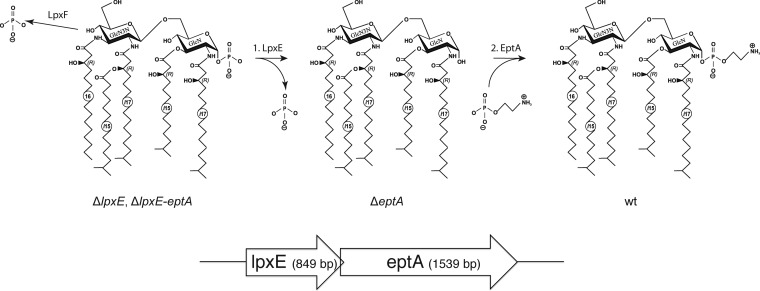
Schematic representation of the proposed enzymatic activity of LpxE, EptA, and LpxF in the biosynthesis of C. canimorsus lipid A (top) and illustration of the *lpxE-eptA* operon (drawn to scale) (bottom) corresponding to *Ccan*_*16960* and *Ccan*_*16950*, respectively.

The genome of C. canimorsus 5 (28) (GenBank accession no. CP002113.1) was analyzed for proteins with high sequence similarity to lipid A-modifying enzymes LpxE and EptA. Our search for a lipid A phosphatase was based on LpxE and/or LpxF sequences from P. gingivalis (1), F. novicida (11), *R. etli* (10), and H. pylori (12, 16) and on all available Bacteroidetes group *pgpB* sequences. Three *lpxE* or *lpxF* candidates were found (*Ccan_16960*, *Ccan_14540*, and *Ccan_06070*) and individually deleted. Interestingly, the gene downstream of *Ccan_16960* (GenBank accession no. YP_004740919.1), *Ccan_16950* (GenBank accession no. YP_004740918.1), was found to have high sequence similarity to *eptA*, coding for a lipid A *P*-Etn transferase. *Ccan_16950* and *Ccan_16960* form an operon, and the two genes overlap by 20 bp (Fig. 2, bottom). *Ccan_16960* has thus been annotated as *lpxE*, an annotation validated by mutagenesis, MS analysis, and impact on endotoxicity and CAMP resistance, as described below. The association of *lpxE* and *eptA* genes reinforces the idea that the two gene products act in the same pathway and suggests that the modification of lipid A that they determine together is essential for survival in the environment of a dog's mouth.

### Predicted lipid A structures in *eptA*, *lpxE*, and *lpxE-eptA* deletion mutants.

EptA has been proposed not to be active on lipid A, in case the 1-phosphate has not been removed before by LpxE (15, 16). Hence, deletion of the lipid A 1-phosphatase LpxE, the enzyme proposed to act first in this two-step mechanism, should result in a lipid A having a 1-phosphate (Fig. 2). Upon deletion of only the second enzyme acting in the pathway, the *P*-Etn transferase EptA, a free hydroxy group at the “reducing end” of the lipid A backbone should result (Fig. 2). This would reflect the fact that LpxE is still active even in the absence of EptA (15, 16). The resulting lipid A in the *eptA* deletion mutant is thus predicted to lack both the 4′ phosphate and the 1-phosphate. In the case of a *lpxE* and *eptA* double deletion mutant, the same 1-phospho lipid A is predicted as for the single deletion in *lpxE* (Fig. 2).

The 1-phospho lipid A variant predicted for Δ*lpxE* and Δ*lpxE-eptA* mutants should be the variant with the highest endotoxicity. In the case of an *eptA* deletion mutant, the free (hydroxy) 4′ and 1 position should result in a very low endotoxic lipid A, as is known from completely dephosphorylated synthetic lipid A analogues (37).

### LpxE and EptA impact on endotoxicity.

**FIG 3:**
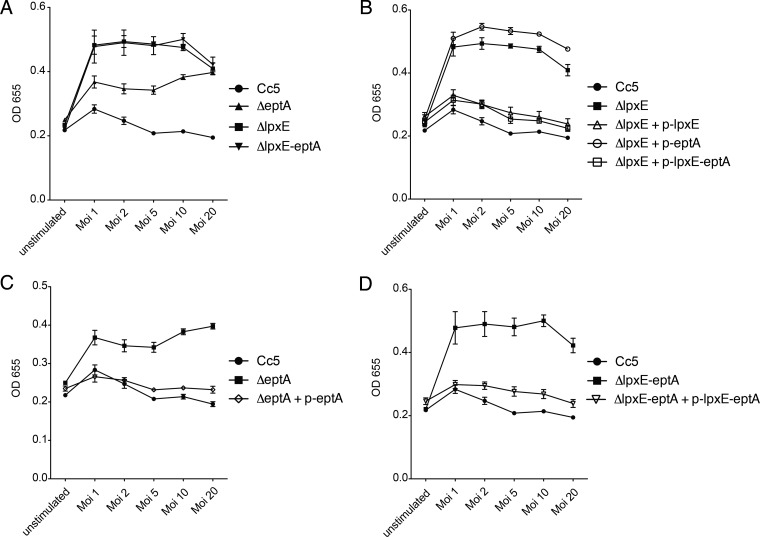
Effect of *lpxE* or *eptA* deletion on endotoxicity. (A) Endotoxicity of heat-killed wild-type C. canimorsus strain 5 (Cc5), Δ*lpxE*, Δ*eptA*, or Δ*lpxE-eptA* bacteria. The indicated multiplicity of infection (Moi) of heat-killed bacteria was assayed for TLR4-dependent NF-κB activation with HekBlue human TLR4 cells. Data were combined from three independent experiments, and the error bars show the standard errors of the means. (B to D) As in panel A but the mutations were complemented in *trans* by the indicated plasmids (p-lpxE, plasmid bearing the *lpxE* gene). All mutations were shown to be nonpolar. Data were combined from three independent experiments, and the error bars show the standard errors of the means.

To study the endotoxic activity after the removal of the 1-phosphate or the addition of an *P*-Etn to the free position 1 of lipid A, we engineered Δ*eptA* and Δ*lpxE* mutations and monitored endotoxicity using a HEK293 cell line overexpressing human TLR4/MD-2/CD14 and a secreted reporter protein (HEKBlue human TLR4 cell line). Activation of this cell line essentially depends on TLR4, and other TLR stimuli may be neglected. Heat-killed bacteria from both mutant strains showed increased endotoxicity compared to wt bacteria, and mutation of *lpxE* had a more severe impact on endotoxicity (Fig. 3A). The endotoxicity of heat-killed C. canimorsus Δ*lpxE-eptA* mutant was identical to that of the C. canimorsus Δ*lpxE* mutant (Fig. 3A).

Complementation of the deleted genes with plasmid-borne genes expressed from the *ermF* promoter restored endotoxicity to the wt level, indicating that none of the mutation was polar (Fig. 3B, C, and D). The Δ*lpxE* strain could be complemented in *trans* with *lpxE* or *lpxE-eptA*, but not with *eptA* alone (Fig. 3B). A slight increase in TLR4 activation of the Δ*lpxE* strain complemented with *eptA* compared to that of the Δ*lpxE* strain was observed (Fig. 3B). This might be explained by transfer of *P*-Etn to other parts of the LPS molecule, as suggested by sequence similarity to the 3-deoxy-d-*manno*-oct-2-ulosonic acid (Kdo) *P*-Etn transferase EptB (38). Finally, the Δ*eptA* strain was complemented with *eptA* or *lpxE-eptA* (Fig. 3C), and the *lpxE-eptA* deletion mutant was complemented with *lpxE-eptA* (Fig. 3D). We conclude from these complementation experiments that the *eptA* and *lpxE* mutations were nonpolar.

To exclude a strong impact on TLR4 activation upon mutation of *lpxE*, *eptA*, or *lpxE* and *eptA* due to various levels of LPS or LPS made accessible by heat killing, we determined the amount of LPS in all strains by Western blot experiments on equal amount of proteinase K-treated bacterial lysates with a C. canimorsus 5 LPS-specific antiserum (see Fig. S1 in the supplemental material). We observed similar LPS band intensities for all strains tested, indicating that LPS amounts present in the bacteria and made accessible by heat treatment are not dramatically changed upon mutation of *lpxE*, *eptA*, or *lpxE* and *eptA*. Notably, a slight size shift of the LPS band was observed for all strains predicted not to exhibit a wt lipid A (Δ*lpxE*, Δ*eptA*, and Δ*lpxE-eptA* mutants and Δ*lpxE* mutant complemented with a plasmid bearing *eptA* [Δ*lpxE* + p-*eptA*]). The migration pattern is altered for all strains predicted not to have the positively charged ethanolamine moiety present, which might explain this observation.

### LpxE and EptA increase resistance to polymyxin B.

**FIG 4:**
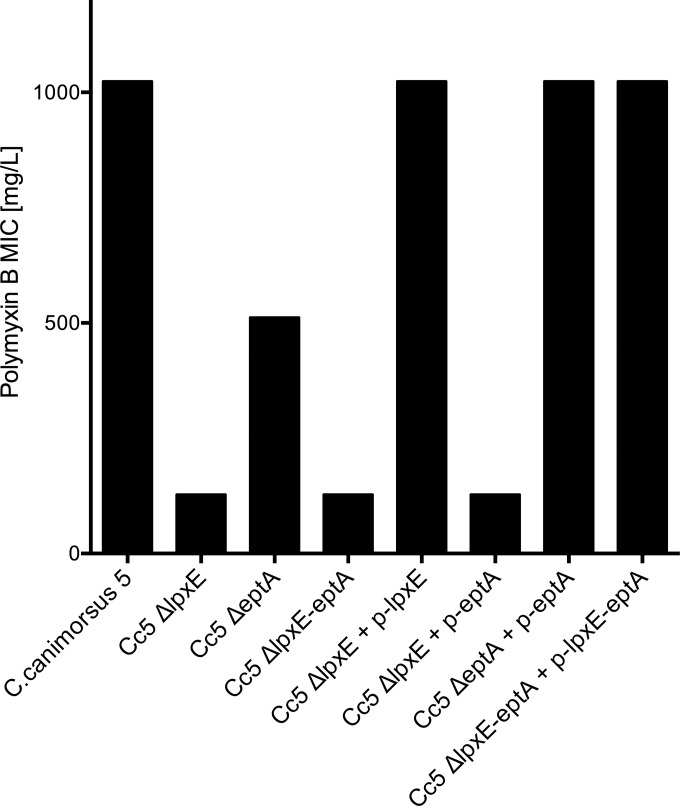
Effect of *lpxE* or *eptA* deletion on resistance to polymyxin B. The MICs of polymyxin B for wild-type C. canimorsus strain 5 (Cc5), Δ*lpxE*, Δ*eptA*, or Δ*lpxE-eptA* and complemented mutants are shown. Data were combined from three or four independent experiments, and the measured MICs were always identical.

Lipid A modifications have been shown to not only affect endotoxicity but also to alter resistance to CAMP such as polymyxin B (10, 29, 39, 40). Hence, we monitored the MIC of polymyxin B for wt C. canimorsus, Δ*lpxE* mutant, Δ*eptA* mutant, and the double-knockout Δ*lpxE-eptA* strains. The wt C. canimorsus was highly resistant to polymyxin B, as it was still able to grow in the presence of 512 mg/liter polymyxin B (MIC ≥ 1,024 mg/liter) (Fig. 4). The MIC decreased to 512 mg/liter for Δ*eptA* mutant bacteria and to 128 mg/liter for the Δ*lpxE* bacteria, showing an increased sensitivity to polymyxin B (Fig. 4). The MIC of the C. canimorsus lpxE-eptA double mutant was the same as that of the single Δ*lpxE* mutant (Fig. 4). The Δ*lpxE* strain could be complemented in *trans* with *lpxE*, but not with *eptA* alone (Fig. 4). The Δ*eptA* strain was complemented with *eptA* (Fig. 4), and the *lpxE-eptA* deletion mutant was complemented with *lpxE-eptA* (Fig. 4). We conclude from these complementation experiments that the *lpxE*, *eptA*, and *lpxE-eptA* mutations were nonpolar.

The *P*-Etn modification at position C-1 thus contributed to the low endotoxicity and polymyxin B resistance of C. canimorsus, as was shown for H. pylori (12). The identical phenotype in endotoxicity and polymyxin B sensitivity of the single Δ*lpxE* and the double Δ*lpxE-eptA* mutants suggests that the *P*-Etn-containing lipid A is synthesized by a two-step enzymatic process similar to that described for H. pylori (15, 16). In H. pylori, lipid A also carries an *P*-Etn group at position C-1, generated in the course of the LPS biosynthesis by removal of the lipid A 1-phosphate by LpxE, followed by transfer of an *P*-Etn residue by EptA from phosphatidylethanolamine to the free reducing end of GlcN(I), where dephosphorylation is necessary for substitution of 1-phosphate with *P*-Etn (12, 15, 16). The nonpolar deletion of *lpxE* in C. canimorsus does not prevent the synthesis of EptA but likely leads to a lipid A with a 1-phosphate group, which would explain the high endotoxicity observed for this strain. Therefore, as in H. pylori, the C. canimorsus EptA seems to accept only the free reducing end of the lipid A backbone generated by the activity of LpxE as a substrate.

The Δ*lpxE* mutation had a more severe effect than the Δ*eptA* mutation, both with respect to endotoxicity and polymyxin B sensitivity. The difference between the two mutants can be explained by the fact that EptA adds a negative charge and a positive charge, whereas LpxE only removes a negative charge. In the two-step mechanism, the Δ*lpxE* mutation would lead to an increase of a negative charge (the unsubstituted 1-phosphate) compared to the wt, while the Δ*eptA* mutation would result in a free reducing end of lipid A compared to the *P*-Etn in the wt. As net negative charges are important for interaction with CAMP as well as with TLR4/MD-2 (41), one would expect Δ*lpxE* to affect endotoxicity and CAMP sensitivity more than Δ*eptA*, which we found. This again supports the two-step enzymatic process of formation of the 1 *P*-Etn.

It is noteworthy that one would expect the mutation of *eptA* not to affect any charge-dependent mechanisms, as no net charge change is expected. C. canimorsus Δ*eptA* bacteria showed increased endotoxicity and decreased CAMP resistance compared to bacteria with wt lipid A, while the lipid A variant predicted for a Δ*eptA* deletion mutant is lacking both the 1 and 4′-phosphate in the lipid A backbone. The C. canimorsus 1-dephospho lipid A in a Δ*eptA* mutant is not expected to be endotoxic at all, as this lipid A species lacks both phosphates, and thus, the negative charges that are important for endotoxicity (41). Still the Δ*eptA* mutation resulted in a small change in polymyxin B sensitivity and a more pronounced change in endotoxicity. This hints at a heterogeneous lipid A population in the Δ*eptA* strain, which could result from a restricted activity of LpxE. Assuming a nonstoichiometric activity of LpxE in the Δ*eptA* strain, both the lipid A containing a free reducing end as well as the 1-phosphate at GlcN(I) should be present. In this case, the 1-phospho lipid A variant could account for the increase in endotoxicity, while its reduced amounts compared to the Δ*lpxE* mutant would explain the higher endotoxicity of the Δ*lpxE* deletion mutant over the Δ*eptA* deletion mutant strain. It might thus be that the accumulation of 1-dephosphorylated lipid A exerts a feedback regulatory effect on the activity of LpxE, preventing full dephosphorylation in the absence of EptA. The fraction of 1-phospho lipid A would thus increase, which would then be responsible for the observed increase in endotoxicity and sensitivity to polymyxin B.

### MS-based structural analysis of *eptA*, *lpxE*, and *lpxE-eptA* deletion mutants.

In order to validate the enzymatic activities proposed for LpxE and EptA and the predicted two-step enzymatic mechanism, we performed MS-based structural analysis of isolated lipid A species of the corresponding deletion mutants. One of the predicted lipid A structures, the 4′- and 1-hydroxy lipid A in Δ*eptA* deletion mutant is devoid of any negative charge and thus not accessible to be analyzed in the negative-ion mode. Therefore, negative- and positive-ion mode MS was run to determine all lipid A variants expected based on the genetic analysis and endotoxic activity, respectively.

**FIG 5:**
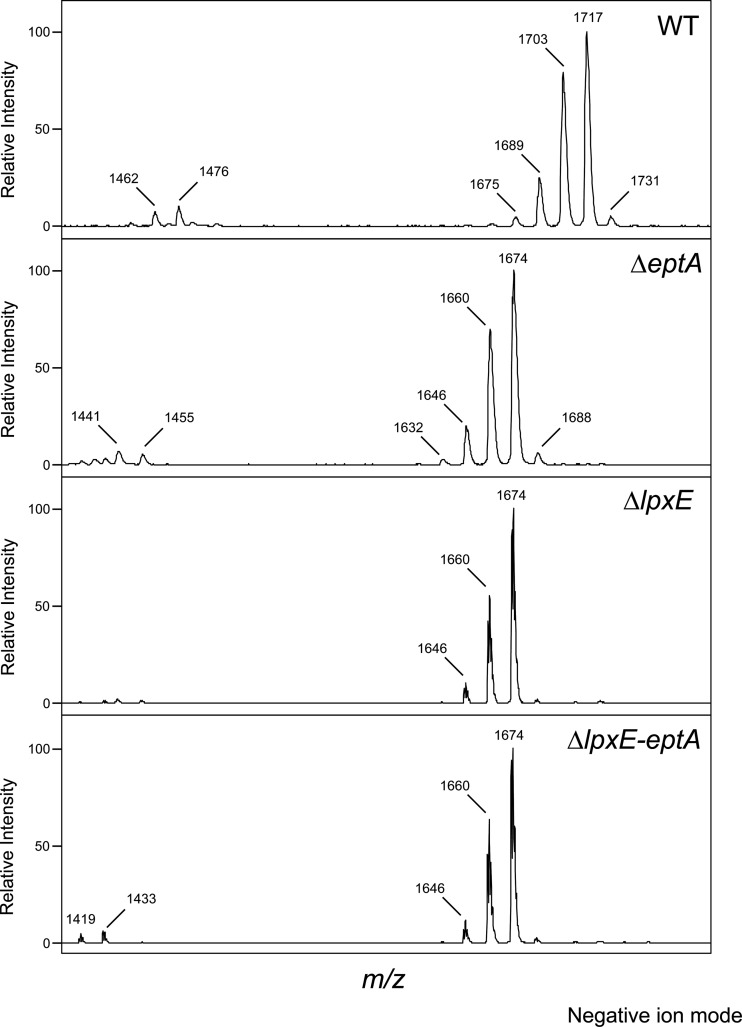
Mass spectrometric analysis of lipid A of the indicated strains as analyzed by MALDI-TOF MS in the negative-ion mode.

In the negative-ion mode, MS analysis confirmed the wt lipid A (calculated *m/z*, 1,716.3; found *m/z*, 1,717) (Table 3 and Fig. 5). Observed mass differences of 14 *m/z* units (*m/z* of 1,731 or 1,703) were assigned to acyl chain heterogeneity. For all samples analyzed by MS, such peak “clusters” differing by Δ14 *m/z* units were found, suggesting that acyl chain heterogeneity was independent of *lpxE* or *eptA* mutagenesis. This is in agreement with our previous data on wt C. canimorsus lipid A (19, 34).

**TABLE 3:** MS analysis and interpretation of lipid A variants in wt and *lpxE*, *eptA*, and *lpxE-eptA* deletion mutant strains

Component or mode and parameter	Value^*a*^ for the component or parameter in the wt	Value^*a*^ for the component or parameter in the following mutant:		
		Δ*lpxE*	Δ*eptA*	Δ*lpxE-eptA*
Components				
GlcN	1	1	1	1
GlcN3N	1	1	1	1
*P*	1	1	0	1
Etn	1	0	0	0
*i*15:0	1	1	1	1
*i*15:0(3-OH)	1	1	1	1
16:0(3-OH)	1	1	1	1
*i*17:0(3-OH)	1	1	1	1
Negative-ion mode				
Calculated *m/z* for the [M-H^+^] ion	1,716.3	1,673.3	1,575.3	1,673.3
Found *m/z* for the [M-H^+^] ion	1,717	1,674	1,674^*b*^	1,674
Positive-ion mode				
Calculated *m/z*	1,764.3^*c*^	1,720.2^*c*^	1,603.3^*d*^	1,720.2^*c*^
Found *m/z*	1,764^*c*^	1,722^*c*^	1,604^*d*^	1,722^*c*^

a 0 indicates that the component is absent, and 1 indicates that the component is present once.

b Ion [M-H^+^] detected in the negative-ion mode of lipid A from the Δ*eptA* mutant was raised from incomplete dephosphorylated lipid A. The major and representative lipid A molecule of this mutant lacks any charged group, and therefore, its pseudomolecular ion [M+Na^+^] could be analyzed only in the positive-ion mode.

c Value for the [M-H^+^+2Na^+^] ion.

d Value for the [M+Na^+^] ion.

In the negative-ion mode, all deletion mutant strains (Δ*eptA*, Δ*lpxE*, and Δ*lpxE-eptA*) showed a main peak at *m/z* of 1,674 (Table 3 and Fig. 5). The 1-phospho lipid A variant predicted for Δ*lpxE* and Δ*lpxE-eptA* mutant strains has a calculated *m/z* of 1,673.3. Hence, Δ*eptA*, Δ*lpxE*, and Δ*lpxE-eptA* deletion mutant strains feature 1-phospho lipid A. While this is the variant expected to occur for Δ*lpxE* and Δ*lpxE-eptA* strains, Δ*eptA* had been predicted to lack the 1-phosphate, thus having a free reducing end for GlcN(I) in the lipid A backbone (calculated *m/z*, 1,575.3). However, due to the lack of a negative charged group, this dephospho lipid A variant cannot be accessed by MS analysis in the negative-ion mode. Nevertheless, the detection of 1-phospho lipid A as well in the Δ*eptA* deletion mutant strain is in perfect agreement with the intermediary phenotype observed in endotoxicity and CAMP resistance (Fig. 3 and 4) and the proposed nonstoichiometric activity of LpxE in the Δ*eptA* strain.

In order to further confirm the postulated enzymatic mechanisms, we performed negative-ion mode MS analysis also on complemented mutants (see Fig. S2 in the supplemental material). The Δ*lpxE* strain could be complemented in *trans* with *lpxE*, as we confirmed the wt lipid A for the Δ*lpxE* strain complemented with plasmid bearing *lpxE* (p-*lpxE*) (calculated *m/z*, 1,716.3; found *m/z*, 1,717). The Δ*lpxE* strain could not be complemented in *trans* with *eptA* alone (Fig. S2), and the resulting strain showed a main peak at *m/z* of 1,674 (Fig. S2), matching the 1-phospho lipid A variant predicted for the Δ*lpxE* strain (calculated *m/z*, 1,673.3). The Δ*eptA* strain was complemented with *eptA* (Fig. S2), and the *lpxE-eptA* deletion mutant was complemented with *lpxE-eptA* (Fig. S2), as in both cases the wt lipid A for these strains was found (calculated *m/z*, 1,716.3; found *m/z*, 1,717).

Additional peaks measured at an *m/z* of 1,755 or 1,769 for the Δ*lpxE* + p-*lpxE* and the Δ*eptA* + p-*eptA* strain are attributed to a minor lipid A variant with two phosphates present and possibly with a classical GlcN-GlcN backbone, known to be present in C. canimorsus 5 (calculated *m/z*, 1,755.209; peak shift of Δ14 *m/z* units due to acyl chain heterogeneity) (19). The two phosphates might either be present as 1-phospho 4′-phospho lipid A or as 1-pyrophosphate lipid A. On the basis of the 1-pyrophosphate species detected in various species (42), we hypothesize that the peaks measured at an *m/z* of 1,755 or 1,769 correspond to a 1-pyrophosphate variant with a classical E. coli type GlcN-GlcN lipid A backbone. The peak measured at an *m/z* of 1,725 for the Δ*lpxE* + p-*eptA* strain is similarly attributed to a bisphosphorylated species in combination with an exchange of 17:0(3-OH) by 15:0(3-OH) (calculated *m/z*, 1,725.186). The detection of these peaks exclusively in complemented mutants and the resulting changes in acyl chain preference and backbone structure, however, should be investigated further.

We conclude from these complementation experiments that the *eptA* and *lpxE* mutations were nonpolar and that in the complemented mutant strains, Δ*lpxE* + p-*lpxE*, Δ*eptA* + p-*eptA*, and Δ*lpxE-eptA* + p-*lpxE-eptA* strains, the wt lipid A is reconstituted.

**FIG 6:**
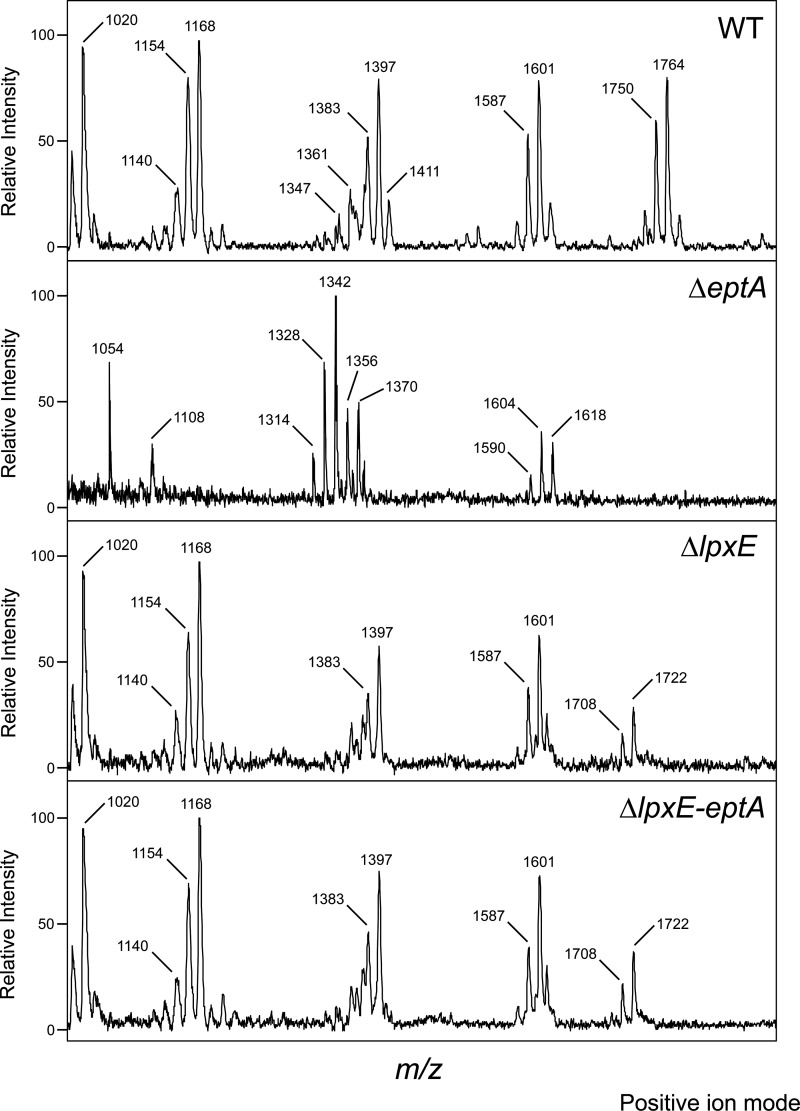
Mass spectrometric analysis of lipid A of the indicated strains as analyzed by MALDI-TOF MS in the positive-ion mode.

In the positive-ion mode, MS analysis confirmed the wt lipid A (calculated *m/z*, 1,764.3; found *m/z*, 1,764) (Table 3 and Fig. 6). Again peak “clusters” differing by Δ14 *m/z* units were found for all samples. The 1-phospho lipid A variant has a calculated *m/z* of 1,720.2 [M-H^+^+2Na^+^] in the positive-ion mode. The main peaks for Δ*lpxE* and Δ*lpxE-eptA* deletion mutants were both found at an *m/z* value of 1,722. This peak was absent in the Δ*eptA* deletion mutant strain. The Δ*eptA* deletion mutant's main peak present at an *m/z* of 1,604 or 1,618 (peak shift of 14 *m/z* units due to acyl chain heterogeneity) corresponded to a free hydroxy group that forms a reducing end in the lipid A backbone and has been calculated to an *m/z* of 1,603.3 or 1,617.3. It is noteworthy that a peak at an *m/z* of 1,601 is found in all samples in the positive-ion mode (and the corresponding peak shifted by 14 *m/z* units). The presence of 1-dephospho lipid A variants even in those mutants which contain the 1-phospho group has been assigned as artifacts well-known to appear from the wt strains due to the acid hydrolysis conditions necessary to liberate the lipid A from the phosphorylated Kdo found in the core of C. canimorsus LPS (19, 34). These conditions obviously lead to a partial dephosphorylation at position 1 of lipid A. The main peak from the 1-hydroxy lipid A cluster for Δ*lpxE* and Δ*lpxE-eptA* deletion mutants was found at an *m/z* of 1,601 and not at an *m/z* of 1,604/1,618 as predicted. We assume that the peak at 1,604 *m/z* found in the Δ*eptA* deletion mutant is identical to peaks at *m/z* of 1,601 found in Δ*lpxE* and Δ*lpxE-eptA* deletion mutants.

According to the proposed model, the major and representative lipid A molecule of the Δ*eptA* mutant lacks any charged group, and therefore, its pseudomolecular ion [M+Na^+^] can be analyzed only in the positive-ion mode. In agreement with this, in the positive-ion (but not the negative-ion) mode of Δ*eptA* lipid A, the pseudomolecular ion [M+Na^+^] was detected. The ion [M-H+] detected in the negative-ion mode of Δ*eptA* lipid A was likely raised from incomplete dephosphorylated lipid A, resulting in 1-phospho lipid A. Notably, the [M-H^+^] ion was not detected for the Δ*eptA* mutant in the positive-ion mode, possibly reflecting its small proportion.

We further performed positive-ion mode MS analysis on complemented mutants (see Fig. S3 in the supplemental material). The Δ*lpxE* strain could be complemented in *trans* with *lpxE*, as we confirmed the wt lipid A for this strain (calculated *m/z*, 1,764.3; found *m/z*, 1,764). The Δ*lpxE* strain could not be complemented in *trans* with *eptA* alone (Fig. S3), and the resulting strain showed a main peak at an *m/z* of 1,722 (Fig. S3), matching the 1-phospho lipid A variant predicted for Δ*lpxE* (calculated *m/z*, 1,720.2). The Δ*eptA* strain was complemented with *eptA* (Fig. S3), and the *lpxE-eptA* deletion mutant was complemented with *lpxE-eptA* (Fig. S3), as in both cases, the wt lipid A for these strains was found (calculated *m/z*, 1,764.3; found *m/z*, 1,764).

Additional peaks measured at *m/z* values of 1,803 and 1,817 for the Δ*lpxE* + p-*lpxE* and the Δ*eptA* + p-*eptA* strain are attributed to a minor bisphosphorylated lipid A backbone variant, probably with a E. coli type classical GlcN-GlcN backbone known also to be present in small amounts in C. canimorsus 5 (calculated *m/z*, 1,802.196; peak shift of 14 *m/z* units due to acyl chain heterogeneity) (19) and that correlates with additional peaks observed in the negative-ion mode MS analysis of these strains.

We conclude from these complementation experiments that the *eptA* and *lpxE* mutations were nonpolar and that in the complemented mutants, Δ*lpxE* + p-*lpxE*, Δ*eptA* + p-*eptA*, and Δ*lpxE-eptA* + p-*lpxE-eptA*, the wt lipid A is reconstituted.

Overall, 1-phospho lipid A was validated as the main lipid A variant in Δ*lpxE* and Δ*lpxE-eptA* deletion mutants. This confirms that LpxE acts as a lipid A 1-phosphatase and further corroborates the two-step enzymatic mechanism in which EptA is active only after LpxE-dependent removal of the 1-phosphate on lipid A. In agreement with endotoxicity and CAMP resistance, both 1-phospho and 1-dephospho lipid A variants were found present in the Δ*eptA* deletion mutant. This validates the function of EptA as lipid A phosphoethanolamine transferase and again supposes a two-step enzymatic activity in which LpxE can dephosphorylate lipid A even in the absence of EptA. However, LpxE seems not to dephosphorylate every lipid A in the absence of EptA, which is reflected by the 1-phospho lipid A species identified in the Δ*eptA* deletion mutant.

The lipid A modification described in this work clearly represents a virulence factor, since it dramatically reduces recognition and killing by the host's innate immune system. However, human infections are rare events and dead ends for C. canimorsus. Thus, we can envision that the lipid A modification most likely evolved as a factor favoring the adaptation of C. canimorsus to its natural niche, the dog's mouth.

## Supplementary Material

Supplemental material
