# Supplementary material for: Modification of the 1-Phosphate Group during Biosynthesis of Capnocytophaga canimorsus Lipid A
Source: Infect Immun. 2016 Jan 25;84(2):550–61. doi: 10.1128/IAI.01006-15 (PMC4730577; doi:10.1128/IAI.01006-15)
Supplement: Supplemental material [file supp_84_2_550__index.html]

Supplemental material 

# Modification of the 1-phosphate group during biosynthesis of *Capnocytophaga canimorsus* lipid A

## Supplemental material

- Supplemental file 1 -

  Fig. S1. Immunoblot analysis of proteinase K-treated wt, Δ*lpxE*, Δ*eptA*, and Δ*lpxE* Δ*eptA C. canimorsus* 5 and complemented mutants. Fig. S2. Mass spectrometric analysis of lipid A of various strains as analyzed by MALDI-TOF MS in the negative ion mode. Fig. S3. Mass spectrometric analysis of lipid A of various strains as analyzed by MALDI-TOF MS in the positive ion mode.

  PDF, 2.5M
