# Supplementary material for: Modification of the 1-Phosphate Group during Biosynthesis of Capnocytophaga canimorsus Lipid A
Source: Infect Immun. 2016 Jan 25;84(2):550–61. doi: 10.1128/IAI.01006-15 (PMC4730577; doi:10.1128/IAI.01006-15)
Supplement: Supplemental material [file IAI.01006-15_zii999091579so1.pdf]

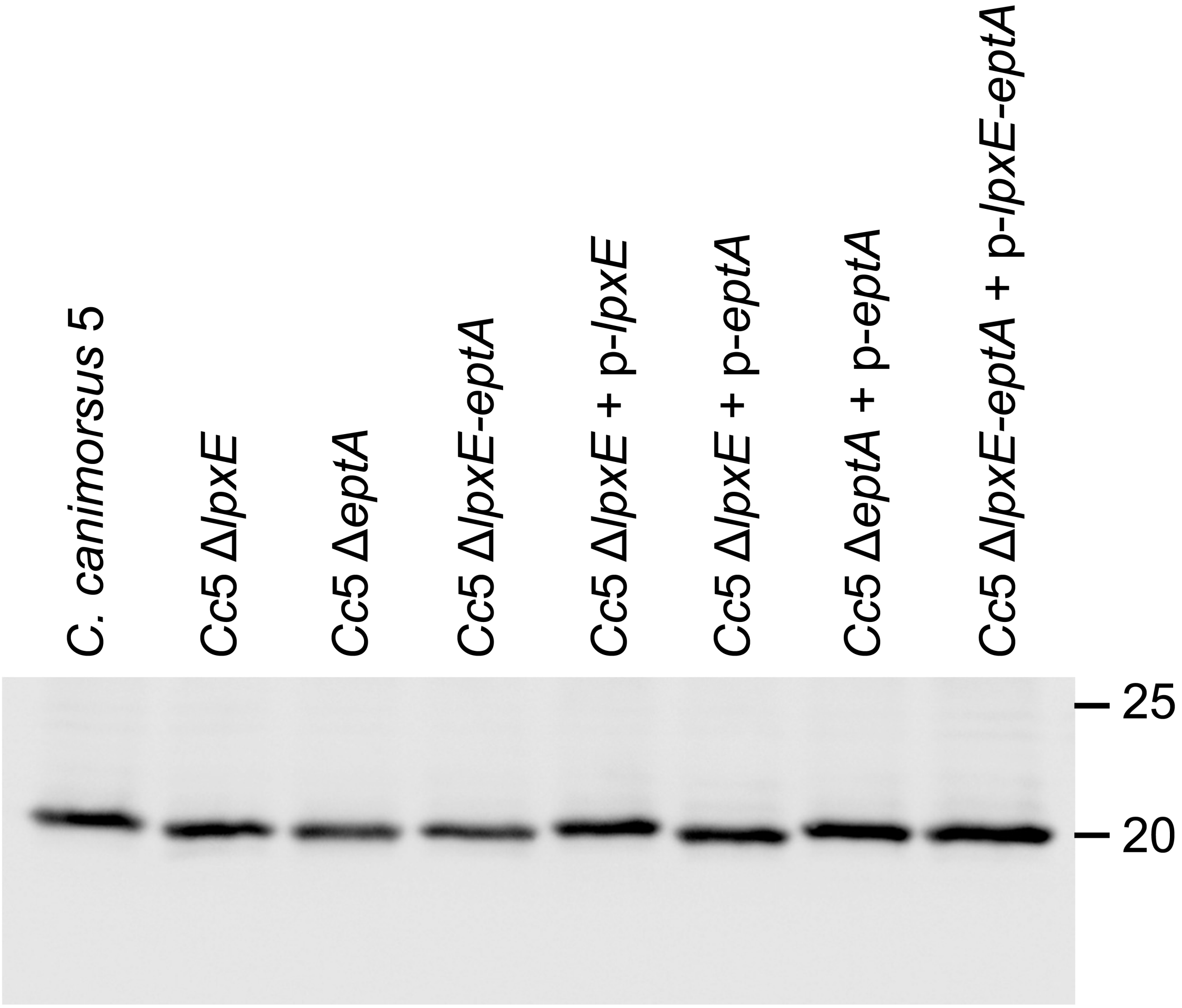

Figure S1. Immunoblot analysis of proteinase K-treated wt *C. canimorsus* 5 (Cc5), Δ*lpxE*, Δ*eptA* or Δ*lpxE-eptA C. canimorsus 5* as well as complemented mutants derived thereof using a *C. canimorsus* 5 LPS recognizing antiserum (Y1C12-absorbed anti-*C. canimorsus* 5 antiserum). As a control, the *C. canimorsus* 5 Y1C12 mutant was loaded. Results shown are representative of four independent determinations. Numbers at the right side of the blot indicate molecular weight in kDa of a protein ladder.

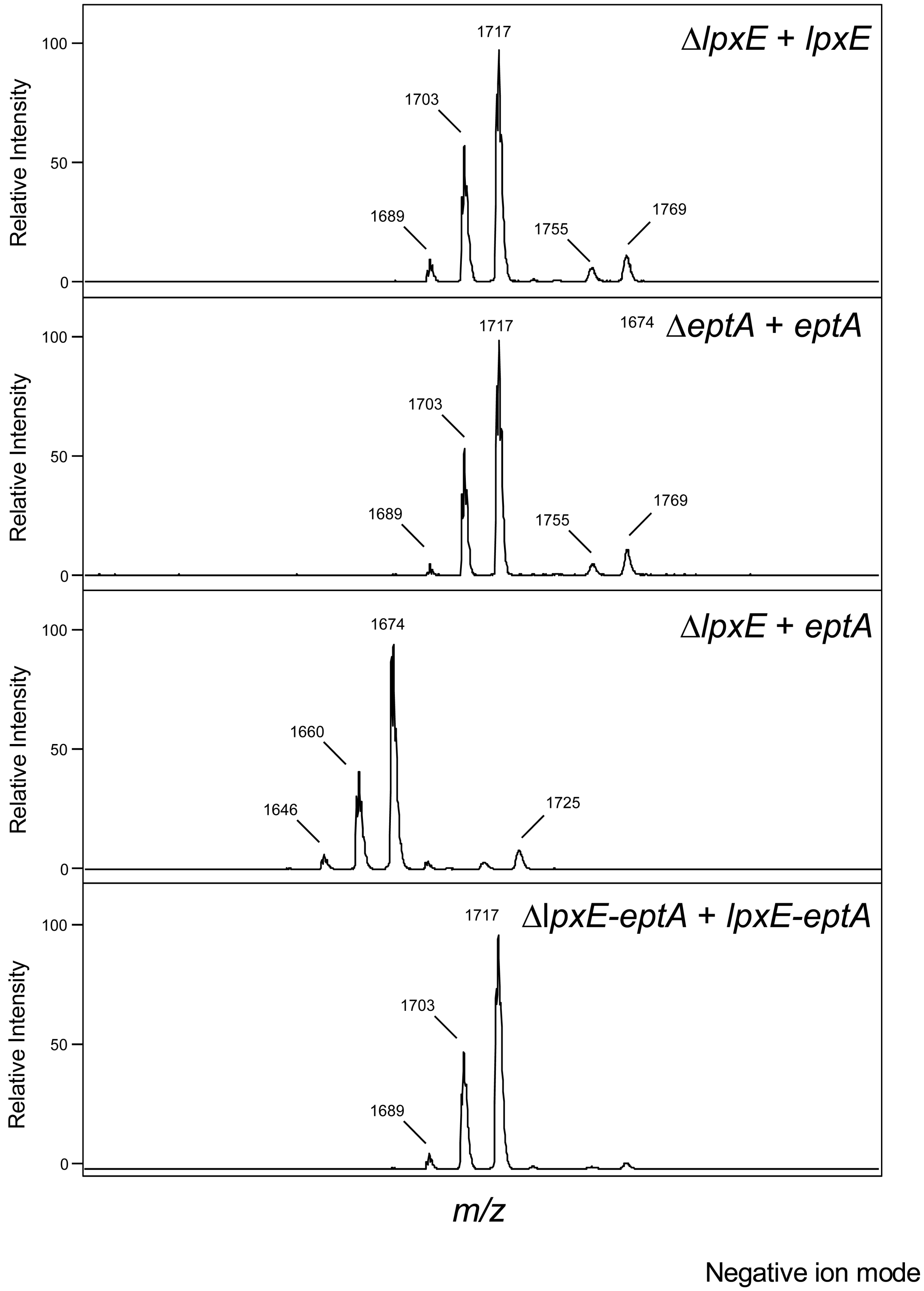

Figure S2. Mass spectrometric analysis of lipid A of indicated strains as analyzed by MALDI-TOF MS in the negative ion mode.

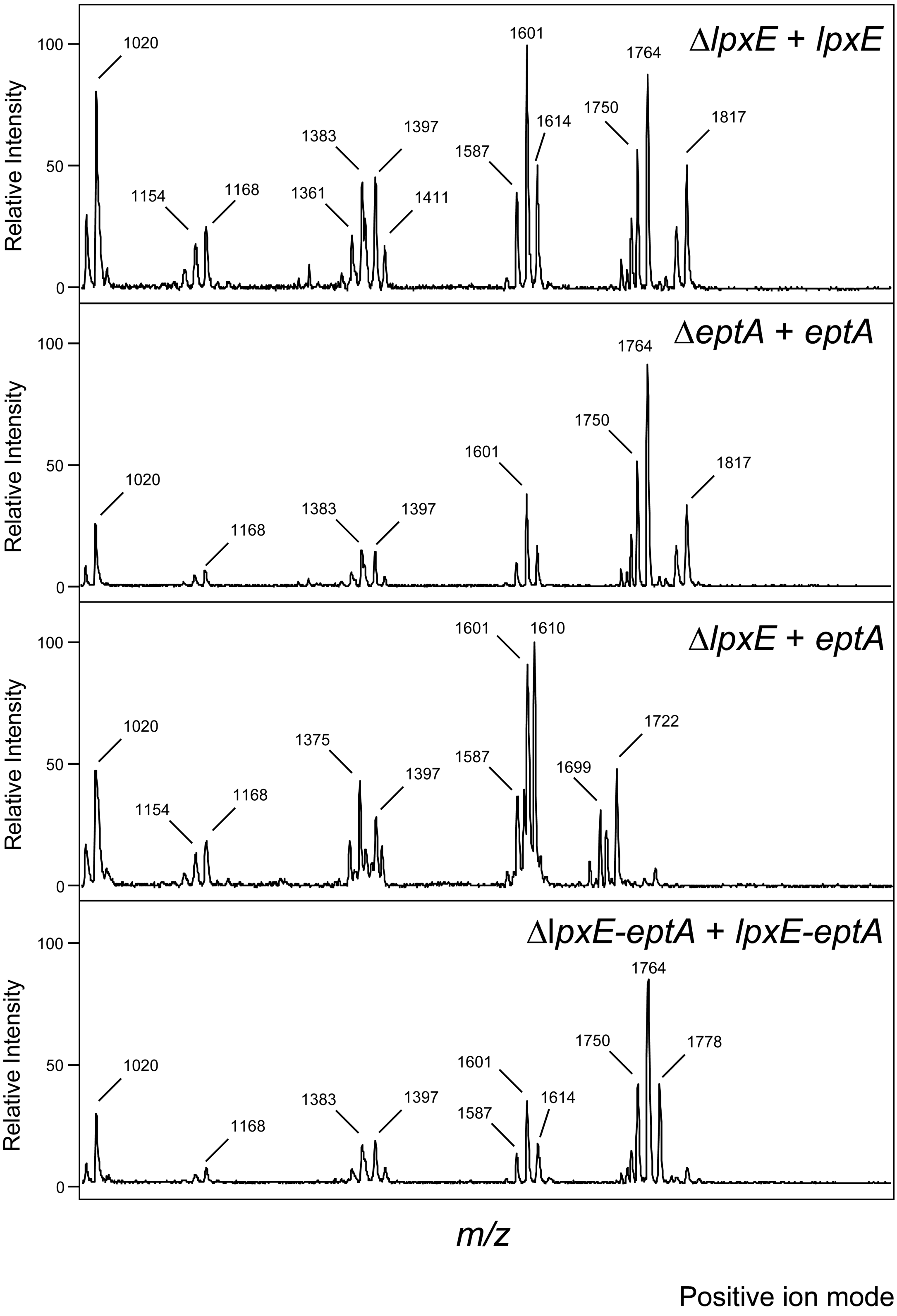

Figure S3. Mass spectrometric analysis of lipid A of indicated strains as analyzed by MALDI-TOF MS in the positive ion mode.
